# Supplementary material for: Thrombophilia Impact on Treatment Decisions, Subsequent Venous or Arterial Thrombosis and Pregnancy-Related Morbidity: A Retrospective Single-Center Cohort Study
Source: J Clin Med. 2022 Jul 19;11(14):4188. doi: 10.3390/jcm11144188 (PMC9316471; doi:10.3390/jcm11144188)
Supplement: Supplementary file 1 [file jcm-11-04188-s001.zip › jcm-1791208-supplementary.pdf]

SUPPLEMENTAL DATA

Supplemental Table S1. Clinical characteristics of cohort patients in accordance to thrombophilia work-up result

**Characteristic**	**Total**<br><br>**n = 3550**	**Negative work-up**<br><br>**n = 2358 (66)**	**Positive work-up**<br><br>**n = 1192 (34)**	**P**
**Venous thrombosis location, n (%)**				<0.001
DVT and/or PE	1791 (50.5)	1194 (50.6)	597 (50.1)	
Superficial vein thrombosis	157 (4.4)	97 (4.1)	60 (5.1)	
Cerebral vein thrombosis	159 (4.5)	121 (5.1)	38 (3.2)	
Splanchnic vein thrombosis	54 (1.5)	44 (1.9)	10 (0.84)	
Retinal/ovarian vein thrombosis	60 (1.7)	48 (2.1)	12 (1.0)	
Arm vein thrombosis	50 (1.4)	36 (1.5)	14 (1.2)	
Other thrombosis*	72 (2.0)	47 (2.0)	25 (2.1)	
**Arterial thrombosis location, n (%)**				<0.001
Stroke or TIA	444 (13)	349 (15)	95 (8.0)	
Peripheral artery disease	47 (1.3)	33 (1.4)	14 (1.2)	
Coronary artery disease	39 (1.1)	31 (1.3)	8 (0.67)	
Retinal artery thrombosis	14 (0.39)	11 (0.47)	3 (0.25)	
Renal artery thrombosis	13 (0.37)	12 (0.51)	1 (0.08)	
Spinal artery thrombosis	6 (0.17)	4 (0.17)	2 (0.17)	
Other thrombosis†	20 (0.56)	15 (0.64)	5 (0.42)	
**Referral for recurrent arterial thrombosis, n (%)**				0.70
Yes	117 (3.3)	92 (3.9)	25 (2.1)	
**Co-morbidities§, ¶, n (%)**				
Diabetes mellitus	138 (3.9)	113 (4.8)	25 (2.1)	<0.001
Arterial hypertension	578 (16)	431 (18)	147 (12)	<0.001
Liver cirrhosis	27 (0.76)	20 (0.85)	7 (0.59)	0.40
Kidney failure	108 (3.0)	91 (3.9)	17 (1.4)	<0.001
Rheumatic disease	218 (6.1)	154 (6.5)	64 (5.4)	0.17
Depression	189 (5.3)	139 (5.9)	50 (4.2)	0.032
Chronic inflammation disease	55 (1.5)	38 (1.6)	17 (1.4)	0.67
Active Cancer	144 (4.1)	111 (4.7)	33 (2.8)	0.006
Dyslipidemia	405 (11)	310 (13)	95 (8.0)	<0.001
Cardiovascular disease**	181 (5.1)	140 (5.9)	41 (3.4)	0.001
Lung disease††	277 (7.8)	189 (8.0)	88 (7.4)	0.50
Neurological disorder‡‡	114 (3.2)	80 (3.4)	34 (2.9)	0.38

Other§§	285 (8.0)	205 (8.7)	80 (6.7)	0.039
**Risk factors for thromboembolism§, ¶, n (%)**				
Heavy Smoking	604 (17)	414 (18)	190 (16)	0.23
Immobilization >4 hours	743 (21)	501 (21)	242 (20)	0.51
Infection	80 (2.3)	58 (2.5)	22 (1.8)	0.24
Estrogen-based treatment	706 (20)	465 (20)	241 (20)	0.72
Pregnancy	153 (4.3)	89 (3.8)	62 (5.4)	0.027
Post-partum	24 (0.68)	19 (0.81)	5 (0.42)	0.18
Intravenous catheter	19 (0.54)	17 (0.72)	2 (0.17)	0.033
BMI > 30 kg m^{-2}	576 (16)	414 (18)	162 (14)	0.002
Trauma	135 (3.8)	81 (3.4)	54 (4.5)	0.11
Surgery	223 (6.3)	170 (7.2)	53 (4.4)	0.001
Extended varicosis	170 (4.8)	119 (5.0)	51 (4.3)	0.31
PFO or other septal defect	218 (6.1)	177 (7.5)	41 (3.4)	<0.001
Atrial fibrillation	38 (1.1)	30 (1.3)	8 (0.7)	0.1
Other¶¶	61 (1.7)	42 (1.8)	19 (1.6)	0.69

Abbreviations: BMI, body mass index; DVT, deep vein thrombosis; PE, pulmonary embolism; PFO, patent foramen ovale; TIA, transient ischemic attack. Categorical values are compared by x^2 test and continuous variables by ANOVA test. *Penis vein thrombosis, muscle vein thrombosis, vena cava thrombosis, kidney vein thrombosis, thrombosis of small vessels (livedo vasculopathy). †Osteonecrosis, mesenteric artery thrombosis, aortic thrombosis, chronic inflammatory disease. §At time of VTE, ATE or pregnancy-related morbidity or at time of consultation in asymptomatic patients. ¶Values were missing for all types of co-morbidities (0.08%) and risk factors such as estrogen-based treatment (0.03%), history of trauma (0.06%), extended varicosis (0.03%). **Cardiomyopathy, cardiac arrhythmia, aortropathy. ††Asthma, obstructive and interstitial lung disease, sleep apnea. ‡‡Epilepsy, parkinson disease, multiple sclerosis. §§Idiopathic erythrocytosis, migraine, Klinefelter syndrome. ¶¶May-Thurner-syndrome, steroid or testosterone treatment.

Supplemental Table S2. Influence of different thrombophilia on treatment decisions

	Total	No influence on therapy	Positive or potential positive influence		Negative influence		P
**Type of thrombophilia, n (%)**	n=3550	n=3050 (86)	Appropriate decision<br>n=211 (6)	Overlooked thrombophilia result<br>n=82 (2.3)	Decision to overtreat<br>n=11 (0.3)	Decision to undertreat<br>n= 184 (5)	
Heterozygous FVL Mutation	714 (20)	589 (19%)	96 (45%)	20 (24)	6 (55)	1 (0.54)	<0.001
Heterozygous PT 20210G>A mutation	193 (5)	138 (5)	39 (18)	14 (17)	1 (9)	0	<0.001
Protein S < 59%	101 (3)	73 (2)	15 (7)	11 (13)	0	0	<0.001
Antithrombin < 70%	52 (2)	26 (0.85)	10 (5)	15 (18)	1 (9)	0	<0.001
Homozygous FVL mutation	48 (1)	28 (0.92)	13 (6)	6 (7)	1 (9)	0	<0.001
Protein C < 69%	28 (0.79)	19 (0.62)	9 (4)	0	0	0	<0.001
Homozygous PT 20210G>A mutation	5 (0.14)	4 (0.13)	0	1 (1)	0	0	<0.001
Antiphospholipid syndrome	119 (3)	47 (2)	50 (24)	21 (26)	1 (9)	0	<0.001

Abbreviations: FVL, factor V Leiden; PT, prothrombin. 12 work-ups could not be categorized due to unclear statement on treatment decision in the clinical report. Categorical values are compared by x^2 test. General influence of thrombophilia testing across manuscript was calculated by combining appropriated decisions and overlooked results.

Supplemental Table S3. Clinical characteristics and prevalence of thrombophilia in cohort patients according to follow-up status

Characteristics	Tested patients<br><br>n= 3550	Follow-up >30 days<br><br>n = 2429 (68)	Follow-up <30 days<br><br>n = 1121 (32)	P
**Age, year, mean (±SD)***	42 (15)	44 (16)	43 (16)	
**Sex, n (%)**				0.49
Female	2118 (60)	1453 (60)	665 (59)	
**Indication for consultation n (%)**				
Arterial thrombosis	583 (16)	479 (20)	104 (9.3)	<0.001
VTE	2343 (66)	1588 (65)	755 (67)	0.08
Pregnancy-related morbidity	120 (3.4)	93 (3.8)	27 (2.4)	0.049
Asymptomatic patients	504 (14)	269 (11)	235 (21)	<0.001
**Provoking factors of VTE†, n (%)**				0.002
Unprovoked VTE	683 (19)	449 (18)	234 (21)	
Provoked VTE, minor risk factor	1242 (35)	827 (34)	415 (37)	
Provoked VTE, major risk factor	415 (12)	309 (13)	106 (9.5)	
**Referral for recurrent VTE†, n (%)**				
Yes	571 (16)	388 (16%)	183 (16)	0.92
**Number of co-morbidities*, n (%)**				<0.001
0	1999 (56)	1228 (51)	771 (69)	
1	814 (23)	590 (24)	224 (20)	
2 or more	737 (21)	611 (25)	126 (11)	
**Number of risk factors for thromboembolism*, n (%)**				<0.001
0	1259 (35)	802 (33)	457 (41)	
1	1189 (33)	837 (34)	352 (31)	
2 or more	1102 (31)	790 (33)	312 (28)	
**Family history of VTE in first-degree relatives†, n (%)**				
Positive	1106 (31)	686 (28)	420 (37)	<0.001
**Family history of VTE in second-degree relatives†, n (%)**				
Positive	523 (15)	347 (14)	176 (16)	0.48
**Type of thrombophilia, n (%)**				
Heterozygous FVL Mutation	714 (20)	481 (20)	233 (21)	0.21
Heterozygous PT 20210G>A mutation	193 (5)	140 (5.7)	53 (4.7)	0.66
Antiphospholipid syndrome	119 (3)	99 (4.1)	20 (1.8)	0.001
Protein S < 59%	101 (3)	74 (3.1)	27 (2.4)	0.42
Antithrombin < 70%	52 (2)	35 (1.4)	17 (1.5)	0.72

Homozygous FVL mutation	48 (1)	35 (1.4)	13 (1.2)	0.61
Protein C < 69%	28 (0.79)	23 (0.9)	5 (0.45)	0.15
Homozygous PT 20210G>A mutation	5 (0.14)	3 (0.1)	2 (0.18)	0.39

Abbreviations: FVL, factor V Leiden; PT, prothrombin; SD, standard deviation; VTE, venous thromboembolism. Categorical values are compared by x^2 test and continuous variables by ANOVA. Risk factors include smoking, immobilization > 4 hours, cancer, central intravenous catheter, infection, estrogen-based treatment, pregnancy, cancer, obesity, trauma, surgery, cancer and its medication. Co-morbidities include diabetes, arterial hypertension, liver cirrhosis, kidney failure, rheumatic diseases, depression, dyslipidemia, lung diseases, neurological disorders, cardiovascular diseases and chronic inflammatory diseases. *At time of VTE, arterial thrombosis or pregnancy-related morbidity or at time of consultation in asymptomatic patients. †Values were missing for provoking factors of VTE (0.08%), referral for recurrent VTE (0.8%), family history of VTE in first-degree (1.3%) and second-degree (1.7%) relatives.
